# Prior infection-triggered flares predict suboptimal biologic response trajectories in psoriasis: A 52-week longitudinal analysis of the SPEECH registry

**DOI:** 10.1016/j.jdin.2026.06.019

**Published:** 2026-07-08

**Authors:** Ning Yu, Xiuxiu Wang, Qin Yang, Yuye Wang, Xinyi Song

**Affiliations:** a Department of Dermatology, Huashan Hospital, Fudan University, Shanghai, China; b Department of Dermatology, Shanghai Skin Disease Hospital, Institute of Psoriasis, Tongji University School of Medicine, Shanghai, China

**Keywords:** biologic therapy, epidemiology, disease activity, infection-related flares, longitudinal outcomes, psoriasis, treatment response trajectories

*To the Editor*: Environmental triggers, such as infections and psychological stress, are commonly implicated in psoriasis flares, but their influence on long-term biologic treatment response remains poorly defined.^1^ Traditional cross-sectional assessments capture disease severity at a single time point and may be influenced by transient, trigger-driven fluctuations.^2^ Here, we used latent class mixed models (LCMMs) to examine the association between prior trigger history and 52-week biologic treatment response trajectories.

We analyzed 831 adults with psoriasis from the Shanghai Psoriasis Effective Evaluation CoHort (SPEECH) registry who initiated biologic therapy,^3^ had baseline Psoriasis Area and Severity Index (PASI) ≥5, complete baseline data, and at least one post-baseline PASI assessment. Baseline variables included demographic and disease characteristics, comorbidities, prior treatment exposure, flare seasonality, and patient-reported trigger histories. Details of eligibility criteria, trigger assessment, and ethical approval are provided in the Supplementary Methods, available via Mendeley at https://data.mendeley.com/datasets/2398c83dw3/1.

LCMMs were fitted to PASI scores collected over 52 weeks to identify distinct treatment response trajectories.^4^ Details of model fitting and multiple imputation are provided in the Supplementary Methods, available via Mendeley at https://data.mendeley.com/datasets/2398c83dw3/1. Associations between baseline characteristics and trajectory membership were examined using univariable and multivariable logistic regression.

Bayesian information criterion values supported a two-class solution, which was also clinically interpretable (Supplementary Fig, available via Mendeley at https://data.mendeley.com/datasets/2398c83dw3/1). Two distinct response trajectories were identified (Fig 1). The “deep response trajectory” (*n* = 721) showed rapid and sustained PASI reduction, while the “suboptimal response trajectory” (*n* = 110) exhibited slower improvement and persistently higher PASI scores through week 52.

**Fig 1:**
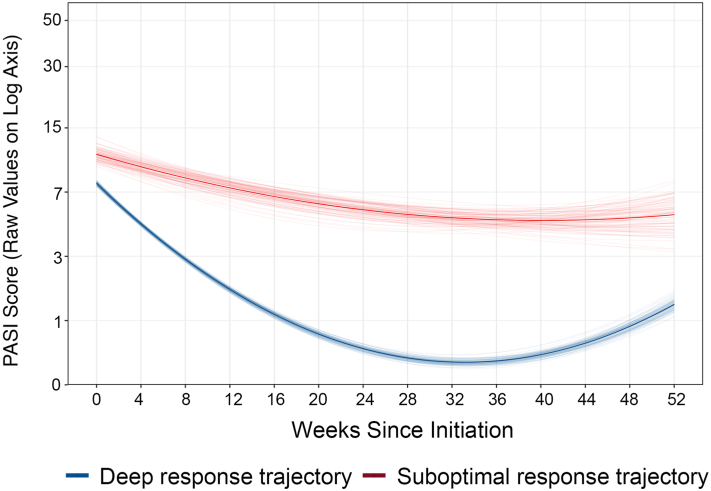
Two 52-week PASI response trajectories after biologic treatment initiation. Latent class mixed models separated patients into 2 longitudinal response patterns. The deep response trajectory (blue) was characterized by rapid, sustained PASI reduction, whereas the suboptimal response trajectory (red) showed a slower decline and persistently higher PASI scores through week 52. *Thin lines* show trajectory estimates from each of the 100 imputed datasets; *thick lines* show the averaged trajectories across imputations. *PASI*, Psoriasis Area and Severity Index.

Baseline characteristics of the study population are presented in Supplementary Table I, available via Mendeley at https://data.mendeley.com/datasets/2398c83dw3/1. After adjustment, infection-related flare history was associated with the suboptimal response trajectory, whereas seasonal flare patterns were not. Other baseline factors, including prior biologic exposure, treatment with adalimumab, higher baseline PASI, higher body mass index, and male sex, were also associated with suboptimal response (Table I). Agreement between optimal trajectory membership and conventional efficacy endpoints was moderate for 75% improvement in PASI (PASI 75) and absolute PASI ≤2 at weeks 12 and 52, but only fair for 90% improvement in PASI (PASI 90), indicating that trajectory membership and single-visit endpoints captured related but different aspects of treatment response (Supplementary Table II, available via Mendeley at https://data.mendeley.com/datasets/2398c83dw3/1). Exploratory subgroup analyses by IL-17A inhibitor status showed a similar direction of association between infection-related flare history and suboptimal trajectory membership (Supplementary Table III, available via Mendeley at https://data.mendeley.com/datasets/2398c83dw3/1).

**Table I:** Baseline factors associated with membership in the suboptimal biologic response trajectory

Variable	Crude OR (95% CI)	*P* value	Adjusted OR (95% CI)	*P* value
Seasonal flare patterns∗		.738		.882
Aseasonal	Reference		Reference	
Multi-seasonal	1.33 (0.70-2.56)		1.33 (0.64-2.78)	
Winter-type	1.32 (0.79-2.30)		1.21 (0.67-2.25)	
Summer-type	1.34 (0.58-2.95)		1.10 (0.44-2.66)	
Trigger histories†				
Infection	2.38 (1.54-3.66)	**<.001**	2.37 (1.43-3.89)	**.001**
Stress	0.81 (0.40-1.51)	.523	0.61 (0.27-1.25)	.184
Other triggers	1.54 (1.01-2.33)	**.044**	1.60 (0.99-2.58)	.055
Baseline PASI score (per 1-point increase)	1.05 (1.03-1.07)	**<.001**	1.06 (1.03-1.08)	**<.001**
BMI (per 1 kg/m^2^ increase)	1.11 (1.05-1.16)	**<.001**	1.10 (1.03-1.16)	**.002**
Psoriatic arthritis	2.09 (1.22-3.48)	**.008**	1.24 (0.64-2.32)	.515
Number of comorbidities‡ (per 1 increase)	1.29 (1.09-1.52)	**.003**	1.16 (0.95-1.40)	.138
Prior biologic exposure	3.70 (2.34-5.79)	**<.001**	3.71 (2.17-6.35)	**<.001**
Male sex	2.68 (1.49-5.24)	**.001**	1.93 (1.01-4.01)	**.047**
Disease duration (per 1-y increase)	1.00 (0.98-1.02)	.965	1.00 (0.98-1.02)	.947
Drug∗		**<.001**		**<.001**
Adalimumab	Reference		Reference	
Ustekinumab	0.63 (0.34-1.20)		0.58 (0.28-1.22)	
Guselkumab	0.27 (0.12-0.59)		0.14 (0.05-0.35)	
Secukinumab	0.18 (0.09-0.35)		0.13 (0.06-0.27)	
Ixekizumab	0.21 (0.10-0.43)		0.14 (0.06-0.31)	

Logistic regression models examined baseline factors associated with the PASI-defined suboptimal response trajectory. Infection-triggered flare history, but not seasonal flare pattern, remained associated with this trajectory after adjustment.

Bold *P* values indicate statistical significance (*P* < .05).

*BMI*, Body mass index; *CI*, confidence interval; *OR*, odds ratio; *PASI*, Psoriasis Area and Severity Index.

∗ Mutually exclusive; *P* values were derived from a global test.

† Trigger histories were patient-reported and not mutually exclusive. Infection-triggered flares referred to psoriasis exacerbation within 1 month after infection; the main specified infection-related triggers were upper respiratory tract infection and periodontitis.

‡ Comorbidities included cardiovascular disease, diabetes, hypertension, non-alcoholic fatty liver disease (NAFLD), hyperlipidemia, and hyperuricemia.

Infection-associated immune activation may contribute to intermittent systemic–cutaneous inflammatory crosstalk and disease worsening despite ongoing biologic therapy.^5^ However, this variable should not be interpreted as proof of a distinct mechanism of biologic nonresponse; it may instead reflect a more difficult-to-control psoriasis phenotype. Limitations include retrospective trigger assessment, recall bias, incomplete infection subtype classification, residual confounding by unmeasured disease activity or host factors, and the observational design, which precludes causal inference. Although exploratory subgroup analyses were performed, the study was not powered to draw definitive conclusions about heterogeneity across biologic classes.

### Declaration of generative AI and AI-assisted technologies in the writing process

AI (Gemini 3) is used for spelling and grammar checks.

## Conflicts of interest

None disclosed.
